# Chikungunya fever in hospitalized children and adolescents: clinical and epidemiological aspects in a region of northeastern Brazil

**DOI:** 10.1016/j.jped.2025.01.010

**Published:** 2025-03-13

**Authors:** Wládia Gislaynne de Sousa Tavares, Roberio Dias Leite, Denise Maria Christofolini

**Affiliations:** a Centro Universitário Faculdade de Medicina do ABC (FMABC), Programa de Pós-Graduação em Ciências da Saúde, Santo André, SP, Brazil; b Universidade Federal do Ceará (UFC), Fortaleza, CE, Brazil

**Keywords:** Arbovirus, Children, Adolescents, Chikungunya fever

## Abstract

**Objective:**

To describe the clinical spectrum of pediatric and adolescent patients infected with Chikungunya.

**Methods:**

Cross-sectional study with patients aged 0 to 17 years hospitalized with a Chikungunya Fever diagnosis in Ceará, in 2017. Data were collected on the clinical manifestations associated with the condition; significant differences were considered when *p* < 0.05.

**Results:**

Fever (100%), erythrodermic rash (90.48%), and arthralgia (52.38%) were the most frequent symptoms. Arthralgia was more prevalent in children older than over five years (86.36%), and irritability and the bullous rash were predominant in children younger than five years (*p* < 0.05). The most predominant non-specific manifestations were: myalgia (28.57%), oral lesions (28.57%), and abdominal pain (26.19%). Neurological complications were observed in 14.29% of the patients, bacterial complications in 11.90%, Kawasaki disease in 4.76%, and one death (2.38% of the population).

**Conclusion:**

Chikungunya fever is a disease that can manifest differently according to age group. The diagnosis must be made early to mitigate possible injuries and complications.

## Introduction

Chikungunya fever is an infectious disease caused by the Chikungunya virus (CHIKV), first identified in Tanzania in 1952.^1^, ^2^, ^3^ Belonging to the *Togaviridae* family,^4^, ^5^, ^6^ CHIKV is primarily transmitted by *Aedes* mosquitoes, with possible vertical and transfusion-related transmission^1^. The disease progresses through acute, subacute, and chronic phases, presenting with high fever, arthralgia, myalgia, rash, headache, nausea, and vomiting, due to viral replication in tissues such as joints, liver, and the central nervous system.^7^, ^8^, ^9^ While rarely fatal, it poses a public health concern due to debilitating pain, prolonged sequelae in adults, and organ impact.^5^^,^^10^

In addition, it is predominantly described in adults during epidemic outbreaks, with a rate of asymptomatic or oligosymptomatic infections ranging from 4% to 28%.^4^^,^^11^ Although infected children present with milder or asymptomatic symptoms compared to adults,^11^ the infection may lead to atypical manifestations such as neurological, cardiovascular, dermatological, ophthalmological, hepatic, renal, respiratory, and hematological complications. Severe complications include shock, arrhythmias, heart failure, encephalitis, Guillain-Barré syndrome, and seizures.^3^^,^^5^^,^^12^

In newborns, symptoms tend to occur between 3 and 7 days of life, ranging from mild to severe cases.^1^^,^^5^ Although myalgia and arthralgia are less common in pediatric patients,^13^ they exhibit a greater diversity of dermatological manifestations, including vesiculobullous exanthema.^4^ Additionally, it is noteworthy that children and adolescents are considered vulnerable groups to infection,^1^ with the potential to develop various complications that require special attention in diagnosis and clinical management, highlighting the importance of age-appropriate approaches for the pediatric population.

CHIKV virus should be suspected in cases of acute fever with polyarthralgia, especially in individuals from endemic areas.^14^ Given the concurrent circulation of dengue, Zika, and CHIKV in these regions, multiplex diagnostic tests are crucial for accurate differentiation and effective management, as co-infections are common during outbreaks.^14^^,^^15^^,^^16^ Diagnosis is confirmed via RT-PCR within the first five days of symptom onset, followed by ELISA serology. However, false positives may occur due to cross-reactivity with other viruses.^1^^,^^5^ Chikungunya typically resolves in 7–10 days, with supportive treatment (hydration, pain and fever control, anti-inflammatory drugs) to prevent complications. Nonsteroidal anti-inflammatory drugs, particularly acetylsalicylic acid, are contraindicated in areas with circulating dengue due to bleeding risks.^1^

This infection is endemic in several regions, with no approved vaccines or antiviral treatments, posing a major global health threat despite ongoing pre-clinical vaccine development. Although mortality rates are low, the virus significantly affects the quality of life and causes economic losses, especially in developing countries. Around 1.3 billion people are at risk,^17^ particularly in Africa, Asia, and the Americas, with climate change potentially accelerating its spread to new areas.^18^ Brazil, particularly in the Northeast region, reports the highest number of cases in the Americas.^4^^,^^6^^,^^7^ From 2013 to 2022, Ceará experienced seven epidemic waves, with the 2017 wave notably impactful.^19^^,^^20^

In the pediatric population, chikungunya represents an increasing threat to children, who often experience additional complications due to the vulnerability of the immune system and a high burden of musculoskeletal symptoms.^1^ Data show an increase in cases among children, with negative impacts on both the school and family environment, as a result of missed activities and the need for ongoing medical care. Pediatric hospitalizations have risen during outbreaks, placing significant strain on healthcare services, and requiring a rapid and effective response. Additionally, the infection leads to high costs associated with medical treatments, consultations, and medications, directly affecting family economics and the public healthcare system.^20^^,^^21^

Given the above, describing the clinical profile of children and adolescents hospitalized for CHIKV in Ceará is essential to understanding disease variations in this age group, considering the regional and epidemiological context. The lack of specific studies on pediatric and adolescent manifestations creates a significant gap in the literature, hindering the development of appropriate management and prevention strategies. Therefore, further research is necessary to enhance early diagnosis, and effective treatment, and to inform region-specific public health policies. This study aimed to describe and characterize the clinical profile of hospitalized children and adolescents with acute CHIKV infection in a reference hospital for infectious diseases in Ceará, Brazil, during the 2017 epidemic.

## Materials and methods

### Design and study population

A descriptive cross-sectional study was conducted with data from 42 pediatric patients diagnosed with Chikungunya Fever at Hospital São José, a reference center for infectious diseases in Fortaleza, Ceará. The study included all pediatric patients admitted with a serological diagnosis of Chikungunya Fever, allowing for the evaluation of a diverse clinical spectrum under varying health conditions.

### Inclusion and exclusion criteria

The study included data from patients aged 0 to 17 years who were hospitalized from March to June 2017 at Hospital São José and were diagnosed with Chikungunya Fever through a positive serological test (ELISA IgM). There were no cases excluded from the study, all cases were analyzed.

Maternal serological or molecular confirmation of chikungunya is not routinely performed in hospitals, particularly during epidemic outbreaks, when resources are focused on pediatric case management. Limited maternal testing prior to 2017 reflects chikungunya's historically low incidence. This study prioritized characterizing clinical and laboratory findings in children and adolescents hospitalized with confirmed serological diagnoses (ELISA IgM).

### Data

The study was carried out through a review of medical records, data were collected specifically at the local study center, thus ensuring better security with the documents and information recorded in these medical records. Epidemiological and clinical variables (symptoms and physical examination) such as sex, origin, age group, and the spectrum of symptoms presented were analyzed.

**Figure 1:**
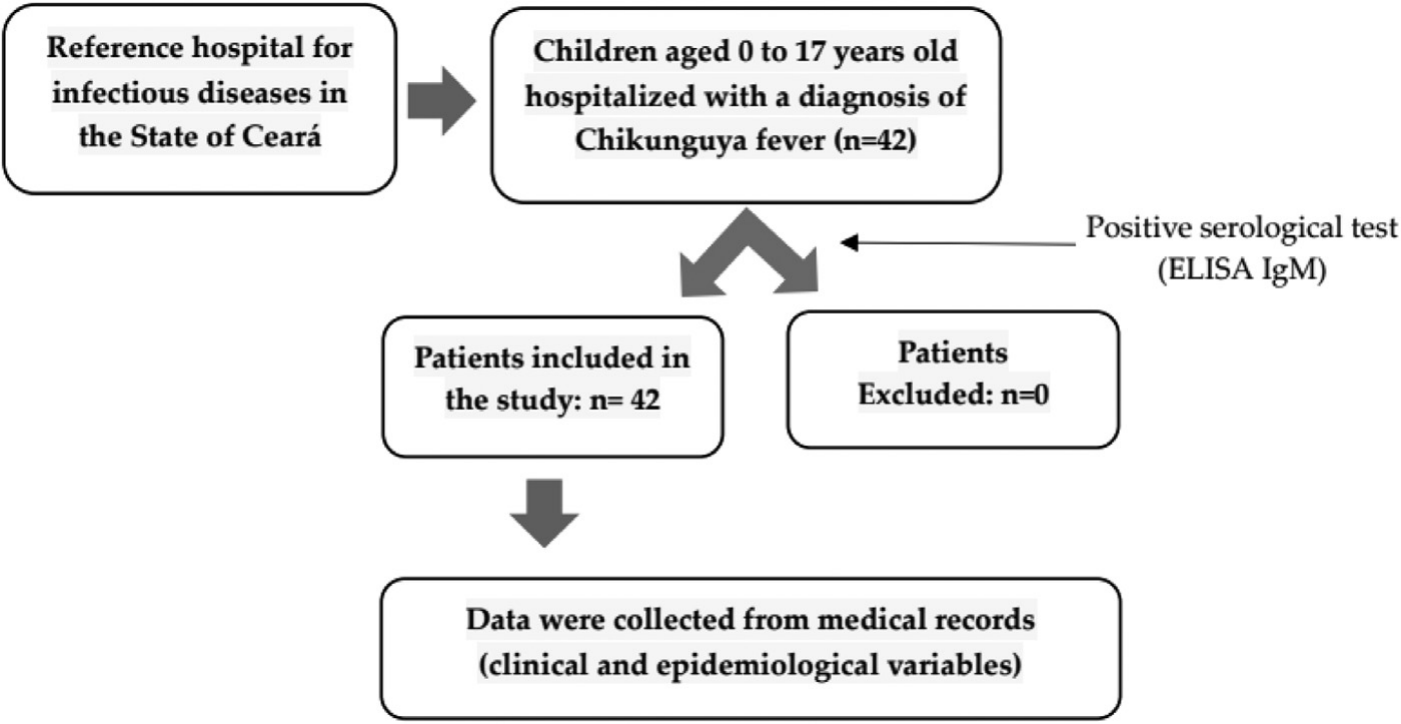
Graphic representation that SU sample collection and data collection.

The graphical representation that summarizes sample collection and data collection is illustrated in Figure 1.

### Statistical analysis

The variables were organized in spreadsheets in the Excel program and subsequently scrutinized using the statistical software EPI-INFOTM version 7.0 (Center for Surveillance, Epidemiology & Laboratory Services, USA), which allowed the data tabulation for statistical analyses. The results were described in absolute (n) and relative (percentages) variables.

To analyze variables, the sample was divided into two groups: children under five years old and those over five. This approach highlighted the high severity and low prevalence of oligosymptomatic cases in infants, as older children can better articulate symptoms like pain intensity. Infants and young children (<2 years) are particularly vulnerable due to immune system immaturity and often exhibit atypical or severe manifestations of infectious diseases, including Chikungunya. Limited communication in this age group complicates identifying subjective symptoms such as arthralgia and headaches. In contrast, older children (>5 years) can describe key symptoms like joint pain, fatigue, and fever, with disease presentations more closely resembling those in adults and fewer oligosymptomatic cases.^22^^,^^23^

These age-based categories were chosen to capture differences in immune vulnerability, clinical manifestations, and the ability to report symptoms, supporting a more precise and targeted characterization of Chikungunya infection profiles.

Comparisons between groups were performed with the Chi-square or Fisher's exact test, and *p* < 0.05 was considered a significant difference.

### Ethical considerations

The research followed all ethical principles in force according to Resolution 466/12 of the National Health Council; the research has approval from the Research Ethics Committee (CAAE: 69,258,617.8.0000.5044) of Hospital São José de Doenças Infecciosas, linked to the Ceará State Health Secretariat.

## Results

Forty-two patients were hospitalized between March and June 2017; the majority (71.43%) came from the state capital, Fortaleza; 52.38% were female, with a median of 5.13 years. Fifty percent were older than five years, and 38.09% of two years or less (Table 1).

**Table 1:** Distribution of patients according to gender, age group, and origin city.

Socio-demographic variables	n (%)
TOTAL	42 (100.00)
Biological sex	
Female	22 (52.38)
Male	20 (42.62)
Age group	
<5 years old	21 (50.00)
>5 years old	21 (50.00)
Origin city	
Fortaleza	30 (71.43)
Caucaia	4 (9.52)
Maracanau	3 (7.14)
Aquiraz	1 (2.38)
Cascavel	1 (2.38)
Itaitinga	1 (2.38)
Maranguape	1 (2.38)
São Gonçalo do Amarante	1 (2.38)

The most prevalent symptoms in the study population are summarized in Table 2. Fever was observed in 100% of cases (*n* = 42), with a median duration of 4 days (range: 2–19 days). Erythrodermic rash occurred in 22 cases, appearing on the first day of fever in 69% (27/39) of patients. Arthralgia was significantly more frequent in children over 5 years old (86.36%, *p* < 0.05), while vesico-bullous rash (91.67%) and irritability (84.21%) were more common in children under 5 years (*p* < 0.05). Joint edema was reported in 11 patients, primarily affecting the knees and ankles, with additional cases of hand and periorbital edema. Pain syndrome was present in all patients younger than two years (*n* = 16).

**Table 2:** Most prevalent symptoms in the pediatric population according to age group.

Symptoms	Age Group	
	0 to 5 years old	>5 years old
	n (%)	n (%)
TOTAL	21 (100.00)	21 (100.00)
Fever	21 (100.00)	21 (100.00)
Erythrodermic rash	19 (90.47)	19 (90.47)
Arthralgia	3 (14.28)	19 (90.47)*
Irritability	18 (85.71)	1 (4.76)**
Pruritus	8 (38.09)	10 (47.61)
Vomit	5 (23.80)	10 (47.61)
Headache	1 (4.76)	12 (57.14)
Vesico-bullous rash	11 (52.38)	1 (4.76)***
Feet edema	10 (47.61)	2 (9.52)
Joint swelling	5 (23.80)	6 (28.57)
Diarrhea	6 (26.57)	4 (19.04)

Statistical significance detected by this Fisher exact test - *p = 0.000001; **p = 0.00000013; ***p = 0.0001.

Non-specific manifestations were also verified, as described in Table 3

**Table 3:** Percentage of patients distributed according to complications and to non-specific clinical manifestations presented.

Complications	n (%)
Neurological	6 (14.29)
Meningoencephalitis	4 (9.52)
Encephaloradiculopathy	1 (2.38)
Acute disseminated encephalomyelitis	1 (2.38)
Bacterial	5 (11.9)
Kawasaki disease	2 (4.76)
Death	1 (2.38)

Manifestations	n (%)

Myalgia	12 (28.57)
Oral lesions	12 (28.57)
Abdominal pain	11 (26.19)
Lymph node enlargement	8 (19.05)
Somnolence	8 (19.05)
Lipothymia	7 (16.67)
Bleeds	6 (14.29)
Ordynophagia	4 (9.52)
Hypotension	2 (4.76)
Non-purulent conjunctivitis	2 (4.76)

It was verified that among the non-specific manifestations, the presence of myalgia and oral lesions stood out in 28.57% of the patients, followed by abdominal pain (26.19%), lymph node enlargement (19.05%), and drowsiness (19 0.05%) (Table 3).

Complications were classified as: neurological in 14.29% (6/42) of patients, bacterial in 11.90% of cases (5/42), Kawasaki disease in 4.76% of cases (2/42), and death in 2.38% of the study population (1/42) (Table 3).

The neurological complications were meningoencephalitis (4/6), encephalo-radiculopathy (1/6), and acute disseminated encephalomyelitis (1/6). The secondary bacterial infections in the sample were pneumonia, acute otitis media, sinusitis, conjunctivitis, and skin infection. The registered death was of a young infant with bullous skin manifestation and secondary bacterial infection (Table 3).

## Discussion

The infection by CHIKV in the study population showed varied symptoms, the most prevalent being fever, erythrodermic rash, arthralgia, and irritability, and non-specific symptoms were also found, mainly: myalgia, oral lesions, and abdominal pain. It was observed that the type of symptoms of the disease might vary with age, such as arthralgia that occurred in a more significant proportion among individuals over five years old, while vesico-bullous rash and irritability were predominantly observed in children younger than five years old; it was also evidenced that all children up to 2 years had pain syndrome. Thus, health professionals need to know the different clinical manifestations of CHIKV infection and their specificities according to the age group to promote an early diagnosis with adequate disease management.

Chikungunya virus poses a significant threat to the pediatric population, as these individuals are more prone to severe forms of the disease due to the specific characteristics of their developing immune system, which impairs defense against viral infections such as CHIKV.^1^ Children under 5 years old, especially those under 6 months, are at greater risk for serious complications, including neurological, cardiac, and dermatological manifestations, often requiring hospitalization.^25^ The increased susceptibility of the central nervous system to viral infections may lead to encephalopathy and disseminated encephalomyelitis, making early diagnosis and proper management more challenging.^1^^,^^25^

Therefore, understanding the specific characteristics of symptoms by age group is crucial for early diagnosis, treatment, and effective monitoring of severe forms of the disease. Each age group presents distinct clinical manifestations, requiring an adapted approach. The vulnerability of children to severe complications necessitates continuous monitoring and the implementation of tailored prevention strategies.

Children exhibit clinical differences compared to adults, with symptoms varying based on age, immune response, viral load, and cytokine levels, despite the pathogenesis mechanism being similar.^1^^,^^2^ Children may be asymptomatic, though this is rare in those under two. The most common symptoms include high fever (>38.9 °C) with sudden onset lasting 1–8 days, cutaneous manifestations (maculopapular rash, pigmentary changes, and bullous lesions), and musculoskeletal disorders (myalgia and arthralgia). Mucocutaneous, articular, hemorrhagic, and neurological manifestations are also observed,^1^^,^^24^ consistent with the findings of the current study.

Other studies report similar findings. Research conducted in Ceará with 14 children, averaging 4.6 years old, found that all had a fever for an average of five days, and 42.8% exhibited joint symptoms. A rash was observed in 92.8% of cases, with 57.1% developing a vesiculobullous rash, most of whom were under a year old.^4^ A study in India at the Karnataka Institute of Medical Sciences (2019), involving 54 patients aged between eight months to 13 years diagnosed with Chikungunya, found that all had fever. Other symptoms included arthralgia (94.44%), myalgia (70.37%), headache (55.56%), vomiting (51.85%), and abdominal pain (51.85%). The study also showed that headache was more common in older children (8 to 13 years) compared to younger ones (3 to 7 years).^2^

The clinical attack rate in children infected with CHIKV may underestimate the disease burden, as some children, especially the youngest, present atypical symptoms, such as undifferentiated fever.^26^ In the current study, younger children (0 to 5 years) showed a higher incidence of irritability and vesiculobullous exanthema compared to older children. These findings align with existing literature, which suggests that infants, particularly those under 3 months, are more likely to experience severe clinical manifestations, such as high fever, marked irritability, and vesiculobullous rashes.^27^^,^^28^

A comprehensive study of 120 infants under 3 months, all hospitalized due to fever, found that 96.2% exhibited irritability and 69.2% developed skin rashes, common signs of Chikungunya infection.^29^ The higher incidence of irritability can be attributed to the difficulty young children have in localizing and communicating pain, especially related to joints or skin. This age group often expresses discomfort through behaviors like crying and complicating diagnosis. Additionally, in this study, children over five years had a higher incidence of arthralgia, a symptom more easily identified in older children. Regardless of age, all children had acute symptoms that were partially or completely resolved by hospital discharge.

A case series study of breastfed infants up to two years old hospitalized for Chikungunya infection in northeastern Brazil concluded that children are a high-risk group for severe and atypical manifestations, including ulcers, vesiculobullous skin lesions, and neurological complications. The main clinical findings were fever, skin manifestations, and irritability. The study highlights the multisystem involvement of CHIKV infection in infants, especially affecting the skin. Irritability in these patients is likely due to pain from skin lesions and osteoarticular involvement.^30^

All patients younger than two years showed pain syndrome, particularly newborns. It is necessary to be alert to the disease in these children considered a risk group, as they have a higher chance of developing severe forms of the disease. In addition, they may be asymptomatic during the first days, with symptom onset from the fourth day on (3 to 7 days), namely: fever, pain syndrome, refusal of breastfeeding, rashes, desquamation, skin hyperpigmentation, and limb edema. Thus, they require daily follow-up until the fever disappears and they observe no signs of severity.^23^

In a recent cross-sectional study, analyzing medical records concerning arboviruses, 159 children were included, 98 suspected cases of CHIKV, and 51 had the diagnosis confirmed. The authors describe the signs and symptoms exhibited in the pediatric population with mild and moderate levels, like findings in adults during an epidemic experienced in a population vulnerable to CHIKV.^15^ The symptoms that the pediatric population in the study showed most frequently were fever (90.2%), arthralgia (76.5%), and rash (62.7%).

Although CHIKV infection is generally considered non-fatal, increased mortality rates were reported during recent epidemics in Pernambuco, Brazil, and Puerto Rico.^5^^,^^31^ Clinical severity follows a U-shaped pattern, peaking in breastfed infants and older adults, with milder manifestations in older children. As no specific treatment exists, management emphasizes supportive care, including hydration, antipyretics, analgesics, and addressing complications when necessary.^22^ Consistent with the published literature, this study reports severe complications, including the death of a one-month-old infant with bullous skin lesions covering over 30% of the body, complicated by secondary bacterial infection and septic shock.

Severe forms in children are more common in neonates, with manifestations including neurological and hemorrhagic complications, as well as myocardial involvement (hypertrophic cardiomyopathy, ventricular dysfunction, pericarditis). Other severe neurological conditions, such as meningoencephalitis, cerebral edema, intracranial hemorrhage, seizures, and encephalopathies, may also occur.^23^ While most CHIKV data come from adult epidemics^1^, recognizing pediatric manifestations is crucial, especially in children under two years, who are at higher risk for severe outcomes. Daily monitoring is advised until the fever subsides and no severe symptoms are present.^23^

Within this context, understanding the clinical manifestations of disease in the pediatric population is crucial for accurate referrals and early diagnosis, considering age-specific symptoms. Healthcare professionals should use diagnostic checklists tailored to age groups, focusing on signs like fever, persistent cough, and respiratory difficulties. In infants, symptoms such as irritability, poor feeding, and breathing issues require attention, while in adolescents, extreme fatigue and gastrointestinal symptoms should be monitored to ensure timely and appropriate follow-up.

Moreover, screening protocols should be implemented in pediatric emergency units, prioritizing the early evaluation of high-risk cases, such as those with a history of respiratory comorbidities or immunosuppression. Epidemiological surveillance actions are critical, with continuous monitoring of cases and clinical manifestations in the pediatric population to allow for the rapid identification and investigation of new viral variants, aiming to mitigate the impact of future outbreaks. The use of electronic notification systems and real-time epidemiological studies can aid in efficient monitoring and the implementation of targeted preventive measures.

## Limitation

The main limitation of this study is the small sample size of 42 pediatric patients, which may impact statistical power and generalizability. However, the sample is representative, as it was drawn from a state referral hospital serving diverse geographic and demographic populations. Data collection occurred during the peak of the 2017 outbreak, enhancing the study's contextual relevance. Additional limitations include the absence of viral load and strain analyses, their correlation with clinical outcomes, and the lack of investigation into comorbidities associated with poorer prognoses. Future studies with larger populations are needed to address these gaps.

Despite these limitations, the use of medical record data, though subject to memory and selection bias, enabled an efficient approach to identifying clinical patterns and gaps in care, providing critical insights into treatment strategies, risk factors, and epidemiological trends. This retrospective analysis proves valuable for supporting public health policies and improving clinical care management.

The CHIKV infection presents various clinical manifestations that differ by age group. In children aged 0 to 17 years, common symptoms included fever, erythrodermic rash, arthralgia, and irritability, along with non-specific signs like myalgia, oral lesions, and abdominal pain. Painful syndromes predominated in two-year-olds, while arthralgia was more common in those over 5, and vesiculobullous rash and irritability were more prominent in children under 5. CHIKV causes global outbreaks, and there are currently no specific treatments or vaccines available. Healthcare professionals must be vigilant in recognizing age-specific clinical manifestations in children to enable early diagnosis, optimize management, and prevent complications and disease progression.

## Conflicts of interest

The authors declare no conflicts of interest.

## References

[1] Martins M.M., Prata-Barbosa A., Cunha A.J. (2020). Arboviral diseases in pediatrics. J Pediatr (Rio J).

[2] SR B., Patel A.K., Kabra S.K., Lodha R., Ratageri V.H., Ray P. (2019). Virus load and clinical features during the acute phase of Chikungunya infection in children. PLoS One.

[3] Cerbino-Neto J., Mesquita E.C., Amancio R.T., Brasil P.E. (2020). Events preceding death among chikungunya virus infected patients: a systematic review. Rev Soc Bras Med Trop.

[4] Beserra F.L., Oliveira G.M., Marques T.M., Farias L.A., Santos J.R., Daher E.F. (2019). Clinical and laboratory profiles of children with severe chikungunya infection. Rev Soc Bras Med Trop.

[5] Dhochak N., Kabra S.K., Lodha R. (2019). Dengue and Chikungunya infections in children: guest editor: bhim S. Pandhi. Indian J Pediatr.

[6] de Lima Cavalcanti T.Y., Pereira M.R., de Paula S.O., Franca R.F. (2022). A review on Chikungunya virus epidemiology, pathogenesis and current vaccine development. Viruses.

[7] Azevedo Rdo S., Oliveira C.S. (2015). Vasconcelos PF. Chikungunya risk for Brazil. Rev Saude Publica.

[8] Moreno-Legorreta M., Tozar-Zamora I., Serrano-Pinto V. (2020). Diagnosis of chikungunya virus infection in Baja California Sur. Mexico. Trop Biomed..

[9] Caglioti C., Lalle E., Castilletti C., Carletti F., Capobianchi M.R., Bordi L. (2013). Chikungunya virus infection: an overview. New Microbiol.

[10] Gordon A., Gresh L., Ojeda S., Chowell G., Gonzalez K., Sanchez N. (2018). Differences in transmission and disease severity between 2 successive waves of Chikungunya. Clin Infect Dis.

[11] Economopoulou A., Dominguez M., Helynck B., Sissoko D., Wichmann O., Quenel P. (2009). Atypical Chikungunya virus infections: clinical manifestations, mortality and risk factors for severe disease during the 2005-2006 outbreak on Réunion. Epidemiol Infect.

[12] Acosta-Reyes J., Rico A., Bayona-Pacheco B., Navarro-Lechuga E., Muñoz F.L., Campo A. (2023). High levels of cardiovascular biomarkers in fatal Chikungunya virus infection. Acta Trop.

[13] Ward C.E., Chapman J.I. (2018). Chikungunya in children: a clinical review. Pediatr Emerg Care.

[14] Staples J.E., Breiman R.F., Powers A.M. (2009). Chikungunya fever: an epidemiological review of a re-emerging infectious disease. Clin Infect Dis.

[15] Gomes P.D., Carvalho R.F., Massini M.M., Garzon R.H., Schiavo P.L., Fernandes R.C. (2022). High prevalence of arthralgia among infants with Chikungunya disease during the 2019 outbreak in northern region of the state of Rio de Janeiro. Front Pediatr.

[16] Silva M.M., Tauro L.B., Kikuti M., Anjos R.O., Santos V.C., Gonçalves T.S. (2019). Concomitant transmission of dengue, Chikungunya, and Zika viruses in Brazil: clinical and epidemiological findings from surveillance for acute febrile illness. Clin Infect Dis.

[17] Nsoesie E.O., Kraemer M.U., Golding N., Pigott D.M., Brady O.J., Moyes C.L. (2016). Global distribution and environmental suitability for chikungunya virus, 1952 to 2015. Euro Surveill.

[18] Tjaden N.B., Suk J.E., Fischer D., Thomas S.M., Beierkuhnlein C., Semenza J.C. (2017). Modelling the effects of global climate change on Chikungunya transmission in the 21st century. Sci Rep.

[19] de Souza W.M., de Lima S.T., Simões Mello L.M., Candido D.S., Buss L., Whittaker C. (2023). Spatiotemporal dynamics and recurrence of chikungunya virus in Brazil: an epidemiological study. Lancet Microbe.

[20] Secretaria de Vigilância em Saúde. Boletim epidemiológico: monitoramento dos casos de dengue, chikungunya e zika até a semana epidemiológica (SE).[Cited 2022 Nov 30]. Available from: https://www.gov.br/saude/pt-br/centrais-de-conteudo/publicacoes/boletins/epidemiologicos/edicoes/2022/boletim-epidemiologico-:vol-53:no 45.

[21] Bueno C.C., Almeida P.R., Castro A.P., Retamero A., Clark L.G. (2017). Aedes Aegypti: economic impact of prevention versus palliation of diseases caused by the mosquito. Value Health.

[22] Ritz N., Hufnagel M., Gérardin P. (2015). Chikungunya in children. Pediatr Infect Dis J.

[23] Saúde Ministério da (2017). Secretaria de Vigilância em Saúde. Chikungunya: Manejo Clínico.

[24] Ernould S., Walters H., Alessandri J.L., Llanas B., Jaffar M.C., Robin S., et al. Chikungunya in paediatrics: epidemic of 2005-2006 in Saint-Denis, Reunion Island. Arch Pediatr. 2008;15:253–62. 10.1016/j.arcped.2007.10.019 18321688

[25] Elzagallaai A.A., Greff M., Rieder M.J. (2017). Adverse drug reactions in children: the double-edged sword of therapeutics. Clin Pharmacol Ther.

[26] Balmaseda A., Gordon A., Gresh L., Ojeda S., Saborio S., Tellez Y. (2016). Clinical attack rate of Chikungunya in a cohort of Nicaraguan children. Am J Trop Med Hyg.

[27] Elenga N., Folin M., Vandamme Y.M., Cuadro-Alvarez E., Long L., Njuieyon F. (2017). Chikungunya infection in hospitalized febrile infants younger than 3 months of age. Pediatr Infect Dis J.

[28] Ferreira M.C., Santos A.K., Marques F.C. (2023). The importance of differential diagnosis of Chikungunya in febrile infants: a new challenge for physicians. CMedUnifoa.

[29] Farias L.A., Pires Neto R.J., Leite R.D (2019). Vesiculobullous exanthema in a 3-month-old child with probable acute chikungunya infection. J Health Biol Sci.

[30] Duarte M do C., de Oliveira Neto A.F., Bezerra P.G., Cavalcanti L.A., Silva V.M., de Abreu S.G. (2016). Chikungunya infection in infants. Rev Bras Saude Mater Infant.

[31] Plotkin S.A. (2019). Chikungunya virus: a back-breaking problem. J Pediatric Infect Dis Soc.

